# Estimated energetic demands of thermoregulation during ancient canoe passages from Tahiti to Hawaii and New Zealand, a simulation analysis

**DOI:** 10.1371/journal.pone.0287290

**Published:** 2023-07-12

**Authors:** Alvaro Montenegro, Alexandra Niclou, Atholl Anderson, Scott M. Fitzpatrick, Cara Ocobock

**Affiliations:** 1 Department of Geography, Ohio State University, Columbus, OH, United States of America; 2 Pennington Biomedical Research Center, Baton Rouge, LA, United States of America; 3 Department of Anthropology, University of Notre Dame, Notre Dame, IN, United States of America; 4 Department of Archaeology and Natural History, Australian National University, Canberra, ACT, Australia; 5 Ngai Tahu Research Centre, University of Canterbury, Christchurch, New Zealand; 6 Department of Anthropology, University of Oregon, Eugene, OR, United States of America; 7 Museum of Natural and Cultural History, University of Oregon, Eugene, OR, United States of America; 8 Eck Institute for Global Health, Institute for Educational Initiatives, University of Notre Dame, Notre Dame, IN, United States of America; 9 Department of Gender Studies, University of Notre Dame, Notre Dame, IN, United States of America; The University of Tulsa, UNITED STATES

## Abstract

Prehistoric colonization of East Polynesia represents the last and most extensive of human migrations into regions previously uninhabited. Although much of East Polynesia is tropical, the southern third, dominated by New Zealand—by far the largest Polynesian landmass—ranges from a warm- to cool-temperate climate with some islands extending into the Subantarctic. The substantial latitudinal variation implies questions about biocultural adaptations of tropical people to conditions in which most of their familiar resources were absent and their agriculture marginal. Perhaps the most basic question, but one which has never been explored, is the extent to which sailing out of the tropics on long-distance colonizing voyages imposed physiological stress on canoe crews and passengers. In this paper we use trajectories of simulated voyages from Tahiti to New Zealand and Tahiti to Hawaii to obtain along-trip environmental parameters which are then used to model the energy expenditure of these long overseas journeys. Results show that travelers to New Zealand are exposed to much harsher environmental conditions, leading to significantly greater in-trip thermoregulatory demands. For both destinations, travelers with larger body sizes exhibit lower modeled heat loss and hence obtain an energetic advantage, with greater gains for females. Such physiological features, notably of Samoans who probably formed the founding population in East Polynesia, may help explain successful voyaging to temperate latitudes.

## Introduction

**Fig 1:**
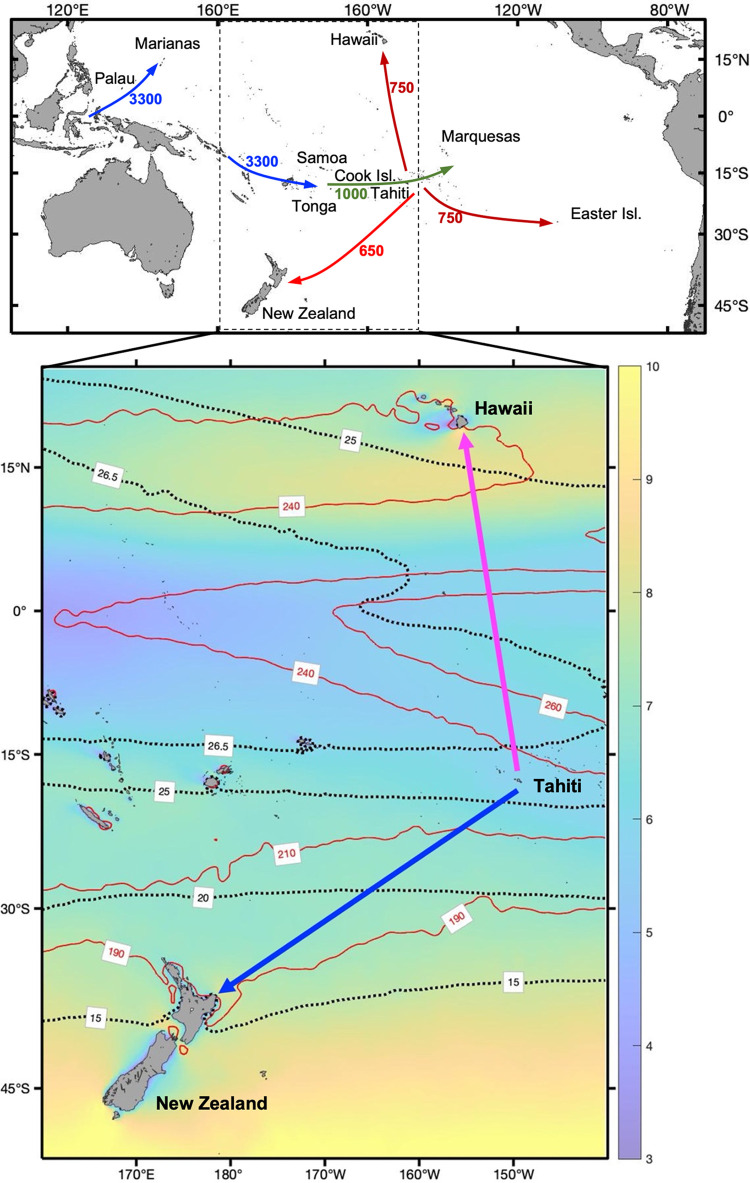
Study area. Top: Map with islands discussed in the text. Dashed rectangle, study area represented below. Arrows represent general migration patterns and adjacent, same-colored numbers provide the approximate date of the event in years before present (B.P.) Bottom, three-year (1987, 1990,1990) mean of parameters adopted by energy balance estimates. Colors, wind speed (m/s); dashed black isolines, temperature; red isolines, downwards solar radiation flux (W/m^2^). Blue and magenta arrows represent approximate path of simulated trips to New Zealand and Hawaii, respectively. See Figs 4 and 5 for along-trip values, and S1 Fig in S1 File for examples of simulated trajectories.

In migrations beginning ca. 3300 B.P., Austronesian speakers from Island Southeast Asia moved northeast into western Micronesia (Palau, the Mariana Is.) and, in the ’Lapita’ migration, southeast through the Bismarck Archipelago as far as Tonga and Samoa (Fig 1). Colonization of central East Polynesia (Society Islands, Cook Islands, Marquesas, etc.) between 1100–900 BP was followed by movement of about 4000 km to the vertices of the Polynesian triangle: Hawai‛i and Easter Island ca. 750 BP and New Zealand ca. 650 BP. These extremely long passages required robust maritime technology in boats, wayfinding, and provisioning, but also exceptional psychological and physiological endurance [1–5].

While the number of people that perished in colonizing Polynesia is unknown, it is likely that there were many failed voyages resulting from storms, loss of bearings, depletion of provisions, and other factors. Unfortunately, available archaeological evidence limits our knowledge about which islands were discovered and when. Failed voyages in which the crew perished will never be known (i.e., Fitzpatrick’s [6] “seafaring paradox”). Extension of colonization from the tropics to more temperate regions also suggests some difficulties. Archaeological evidence from the ‘mystery islands’, those abandoned at contact (e.g., Norfolk, Raoul, Pitcairn, and Henderson), indicates increasing challenges to long-term survival of populations as colonization reached the subtropics and beyond as far as the Subantarctic islands [7]. Restriction in resource availability was a major factor [8] but one probably exacerbated by pressures on voyaging capability.

As such, the study of prehistoric Polynesian colonization involves consideration of environmental, technological, and social factors that shaped the remoteness (relative duration of passages) and isolation (frequency of contact) of islands [7]. Amongst those factors, migration timing [9, 10] and its social imperatives, environmental variation in sailing conditions [6, 11], and canoe sailing capabilities [12] have been discussed recently. However, with few exceptions [13], one particularly fundamental factor has been missing from the discussion; physiological stress experienced by Polynesians at sea. Exposure to heat, cold, wind, spray and rain would have affected human thermoregulation as voyagers travelled through warmer areas close to the Equator and ventured into latitudes from 20˚N to 50˚S where air and sea temperatures were progressively cooler. These overseas ventures would have demanded high levels of physical activity with a relatively limited source of sustenance, creating the potential for a substantial negative energy balance in which voyagers were burning significantly more calories than they were consuming. We contend that, alongside capability in long-distance sailing, navigation, and victualling, challenges in regulating body temperature, maintaining a high energy output, and managing with limited resources helped shape the relative remoteness and isolation of extra-tropical islands in Polynesia as well as morphological variation observed among modern Polynesian populations.

Selective pressures related to thermoregulatory demands associated with exposure to the oceanic environment during voyaging or routine utilization of nearshore resources could have influenced the morphology of Polynesian people. Anthropological history on how body shape and size vary by environment dates back to ecogeographical rules established, notably, by Bergmann [14] and Allen [15]. Bergmann’s rule states that in endotherms, body surface area-to-volume ratio will decline with decreasing temperature (i.e., a greater body mass relative to surface area in cold climates increases metabolic heat production and reduces its loss). Allen’s rule predicts shortened limb segments in cold climates, again to reduce overall body surface area. Physiological evidence linking body shape, size, and thermoregulatory capacities among humans, as defined by the ecogeographical rules, is lacking, but observational evidence suggests they do largely conform to these rules [16, 17]. For example, larger body sizes, typically with greater adiposity and muscle mass, are seen among cold climate populations such as the Inuit [18, 19]. Interestingly, such “typical” cold climate body shapes and sizes are also observed among modern Polynesian populations [20, 21]. Exposure to low temperatures experienced at sea may explain the potential mismatch between Polynesian phenotypes and the ecogeographical rules observed among cold and warm climate populations [22] Proponents of the thrifty genotype hypothesis [23, 24] suggest that cold exposure [13, 25] and/or limited resources during periods of long-distance ocean voyaging could produce selective pressures in these populations leading to large, heat-saving, and (per kilogram) energy-saving body sizes.

Although providing an explanation of the mismatch between Polynesian phenotypes and their tropical environments, the thrifty genotype hypothesis has been criticized for failing to consider archaeological and biological data. The basic tenet of this hypothesis is that early exploratory voyages led to accidental rather than planned settlement. However, evidence of trade and close interconnectedness between Melanesian and Western Polynesian islands can be traced back to 3000 BP through Lapita pottery and obsidian items. The rapid expansion of these artifacts across the southwestern Pacific suggests exchanges between remote islands for extended periods [26, 27] that contradict the accidental voyaging assumption. Furthermore, no “thrifty” genes predisposing individuals to lower energy expenditures, larger body sizes, and greater risks of type II diabetes mellitus [28] have been identified. By itself, the thrifty genotype hypothesis cannot explain the discrepancies between Polynesian morphologies and the Allen and Bergmann rules.

Recent findings of active brown adipose tissue (BAT) in Samoans, provide evidence for the potential role of this thermogenic trait in the migratory success of Polynesians [29, 30]. BAT, a hyper-vascularized heat generating type of fat is a source of non-shivering thermogenesis, allowing hibernating mammals and humans to produce body heat in dropping temperatures [31]. BAT is believed to play an important role in human cold adaptation as it is activated at greater rates in groups indigenous to cold environments compared to those lacking innate physiological protection against chronic cold exposure [32]. BAT likely also contributes to cold acclimatization as noted through seasonal increases in BAT thermogenesis in temperate climate populations [33]. Evidence of BAT activity in Samoan adults suggests BAT may be found across populations, independent of environmental temperature, and may have provided Polynesians with protection against the cold during initial explorations of the Pacific [29]. It must be noted that current understanding of BAT activity rates in adult humans is largely limited to cold [33, 34] and temperate climate populations [35, 36] and only limited studies provide evidence of BAT activity in warm climate populations [29, 30, 37]. The potential thermogenic protection offered by BAT activity during cold exposure experienced at sea may contribute to increased energy expenditure during settling voyages [29, 34, 38]. Until further evidence of BAT activity among warm-climate populations, it remains, however, unclear how much BAT activity contributed to the energetic demands of those trips.

Here we explore these issues using environmental data with simulated ocean voyages and a body energy balance model to calculate values of resting energy balance (*EB*), defined as the difference between basal metabolic rate (BMR) and heat loss flux (L). We adopt *EB* as an estimate of thermoregulatory energy demand to better understand the effort involved in reaching different islands in Polynesia. We analyze *EB* differences experienced by female and male travelers voyaging from Tahiti to Hawaii and from Tahiti to New Zealand (Fig 1), and how differences in traveler body size influence trip *EB*. Estimates are performed assuming both voyages were carried out under similar technological conditions, particularly in terms of individual protection from the elements.

We show that the high metabolic thermoregulatory demand in response to environmental exposure played a role in limiting movement beyond the tropics by decreasing bodily comfort in ways that could not be readily ameliorated, and ultimately by survivability at higher latitudes. We also show that the more efficient thermoregulation of larger-bodied voyagers results in relatively smaller heat loss rates and significant energetic advantages and that these are greater for females. Our *EB* estimates can serve as a foundation for more comprehensive modeling of prehistoric human physiology in oceanic voyaging, with future iterations including estimates of physical activity, caloric intake, brown adipose tissue, and the interaction between thermoregulation, activity, clothing and shelter. Current results suggest that the earlier discovery of Hawaii than New Zealand, despite their similar distances from central East Polynesia, and similar late discovery of other extra-tropical islands, is consistent with relative environmental discouragements to long-distance traveling in colder seas. These include greater wave heights, cloud cover, and storminess. Experience in coping with these and, most fundamentally, with the bodily discomforts of cold outlined here, may have played an important role in shaping Pacific seafaring and colonization patterns and ultimately led to an unknown number of unsuccessful voyages or settlement extinction on some islands in temperate zones.

Below, we outline the parameters and variables used in constructing the voyaging simulation model. We then discuss the equations used to estimate energy balance within different body types (e.g., female and male) during these voyages. Lastly, we present results concerning how different environmental conditions would have affected thermoregulation on simulated trips to both Hawaii and New Zealand from Tahiti.

## Materials and methods

### Voyage simulations

In the adopted voyage simulation model [39, 40], vessel displacement is given by the sum of water surface current and sailing induced velocities. Sailing velocity is a function of destination, wind velocity, and vessel type. Experiments adopt sailing performance parameters from the replica Polynesian sailing canoe “Nalehia” [41]. These are generous parameters given the probability that sailing technology during the East Polynesian migration era was significantly less capable than is commonly assumed [42]. Wind and current data are daily averages obtained from the ERA5 and ORAP5 climate reanalysis respectively [43]. As in all reanalysis sets, the values adopted here constitute a blend of observations and model output that can be understood as numerical model output periodically “corrected” by observations or observations interpolated in space and time by a numerical model. The original set consists of 6-hourly values distributed over a regular 1˚×1˚ spatial grid, resulting in about 100 × 100 km spatial resolution at the equator. Ocean surface current data came from the ECMWF ORAP5 ocean reanalysis. It consists of daily values with spatial resolution of 0.25° × 0.25° [43]. Prior to adoption by simulations, all data are interpolated into daily values with 0.25° × 0.25° spatial resolution. To account for interannual variability, trips are simulated for years with neutral (1990), El Niño (1987), and La Niña (1999) conditions. During each of these years, 16 trips depart Tahiti every five days, eight of those targeting the southern coast of Hawai’i and the other eight the NE coast of Te Ika-a-Māui (S1 Fig in S1 File). Vessel displacement is calculated every six hours for 50 days or until arrival at target. A total of 1752 (8 × 73 × 3) trips are simulated for each destination.

### Resting Energy balance (*EB*)

#### Input data

Trip energy balance estimates adopt hourly, along-trip-trajectory values of surface wind speed, surface air temperature, and downward solar (shortwave) radiation at the surface. The solar radiation data account for the effects of cloud cover and include direct and diffuse radiation. All data have 0.25˚ × 0.25˚ spatial resolution and are from the same ERA5 ECMWF reanalysis project [43] used in simulated trips. They cover the same three years of the voyage simulations, but ERA5 hourly products are used.

#### Model equations

Resting energy balance, *EB*, is given by the difference between Basal Metabolic Rate (*BMR*) and the rate of net body heat loss (*L*), with all values in Watts (W):

EB=BMR−L (1)

*BMR*, is sex dependent and calculated with a version of the “Oxford Equations” derived from a sample containing 13.9 thousand individuals from 23 distinct ethnicities reported in 174 papers published between 1914 and 2001 [44]:

BMR=kcd2W(13.1BM+558) (2A, female)

BMR=kcd2W(16BM+545) (2B, male)

*BM* is body mass in kg. *Kcd*2*W* = 0.04843 and is used to convert the second term on the right-hand side of 2A and 2B from kilocalories per day (kcal/d) to W.

The equation for net heat loss expands on the expression by Sørensen [45] as adopted by Rodríguez et al. [46], and is given by:

$$L=L_{d}+L_{w}-Sa$$ (3)

*L*_*d*_ and *L*_*w*_ are heat loss over the dry and wet fractions of the body respectively. *Sa* is shortwave radiative energy absorption and represents a heat gain, hence the negative sign.


$$L_{d}=(1-wfr)\left(\frac{BSA(T_{c}-T_{a})}{R+\frac{1}{C}}\right)$$ (4A)

$$L_{w}=(1.5*wfr)\left(\frac{BSA(T_{c}-T_{a})}{R+\frac{1}{C}}\right)$$ (4B)

The expression in large brackets shared by 4A and 4B is the original [45] formulation for heat loss and accounts for loss from dry surfaces due to emission of longwave radiation, conduction, and convection. *T*_*a*_ is ambient temperature, in K, and *T*_*c*_ body core temperature (set in our model to 310.15 K, or 37˚C).

*BSA* is body surface area, in m^2^, and a function of BM and height in cm (*H)*:

$$BSA=0.024265H^{0.3964}*{BM}^{0.5378}$$ (5)

*R* is thermal resistance, in m^2^K/W, of all isolating layers, including body fat, and given by the sum:

$$R=\sum_{i=1}^{n}\frac{s_{i}}{k_{i}}{cfr}_{i}$$ (6)

with *s*_*i*_, *k*_*i*_ and *cfr*_*i*_ being thickness (in m), thermal resistance (in W/mK), and fraction of body covered by isolating layer *I*, respectively. The model accounts for two isolating layers: subcutaneous body fat (SCF) and clothing. For SCF *cfr* = 1; k = 0.2 W/mK [46]; and *s* varied in different estimates from 0.005 m to 0.0075 m. Isolation due to clothing is represented by a layer comprised of some type of bark cloth garment with k = 0.0357 W/mK; s = 0.0012 m; and *cfr* varying in different estimates from 0.25 to 0.50. Adopted *cfr* values aim to provide a reasonable range of body clothing coverage. Based on standard methods for allocation of surface area percentage to different body parts [47]. A 0.25 *crf* could be associated, for example, with a cape covering the chest, upper back, and shoulders, while a 0.5 *crf* value could mean coverage of the whole torso plus arms or whole torso plus thighs. Clothing thermal resistance and thickness values come from *Ficus natalensis* bark cloth [48].

*C*, heat loss due to convection in W/m^2^, is a function of wind speed in m/s (*WS*) and given by:

$$C=5+3.95{WS}^{0.6}$$ (7)

The adopted reanalysis provides wind speeds at 10 m elevation, which tend to be higher than speeds closer to the surface, leading to a potential overestimation of convective heat loss. Prior to its use in Eq 7, wind data are adjusted to represent values at 1.5 m elevation by:

$${WS}_{z1}=\frac{{WS}_{z}}{{\frac{z}{z1}}^{P}}$$ (8)

Where z and z1 are elevations of the original and adjusted data, set here to 10 m and 1.5 m respectively; *WS*_z1_ and *WS*_z_ are wind speeds at elevations z1 and z. *P* = 0.11 and is empirically derived for the height dependency of wind speed over the ocean [49]. The 1.5 m value is adopted as representative of the elevation occupied by someone traveling in a Polynesian sailing canoe.

*Wfr* is the average fraction of the body assumed to have remained wet during trips. The increase in latent heat loss due to presence of water on clothing or skin is not accounted for in the original Sørensen [45] expression. Based on direct heat loss measurements over dry and wet skin [50], our model increases heat loss over the wet fraction of the body by 50%. Estimates make use of *wfr* values of 10% and 20%. These are averages in time so that, for example, a traveler with 40% of their body wet for 12 hours a day and who remained completely dry for the other 12 hours would have a *wfr* of 20%. The *EB* model only accounts for dry and wet body surfaces and makes no distinction between different types or levels of “wetness” (for example, wet due to sea spray versus wet due to precipitation).


Sa=BSA*ifr*S(1−α) (9)

Where *S* is incoming solar radiation flux in W/m^2^; *ifr* is illuminated fraction of the body, or fraction of the BSA receiving sunlight, with estimates calculated from *ifr* = 0.3 and *ifr* = 0.4; *α* is skin albedo, or the fraction of incident sunlight reflected by the skin, so that 1-*α* is the fraction of incident sunlight absorbed by the body. Adopted *ifr* values represent estimates of the illuminated body fraction of travelers that are sitting down and receiving radiation on head, neck, and back (*ifr* = 0.3) or on the head, neck, chest, and abdomen (*ifr* = 0.4) [47]. *EB* is calculated using *α* values of 0.3 and 0.25 in order to cover the range of skin *α* observed in present-day low-latitude populations [51, 52].

#### Body types

**Fig 2:**
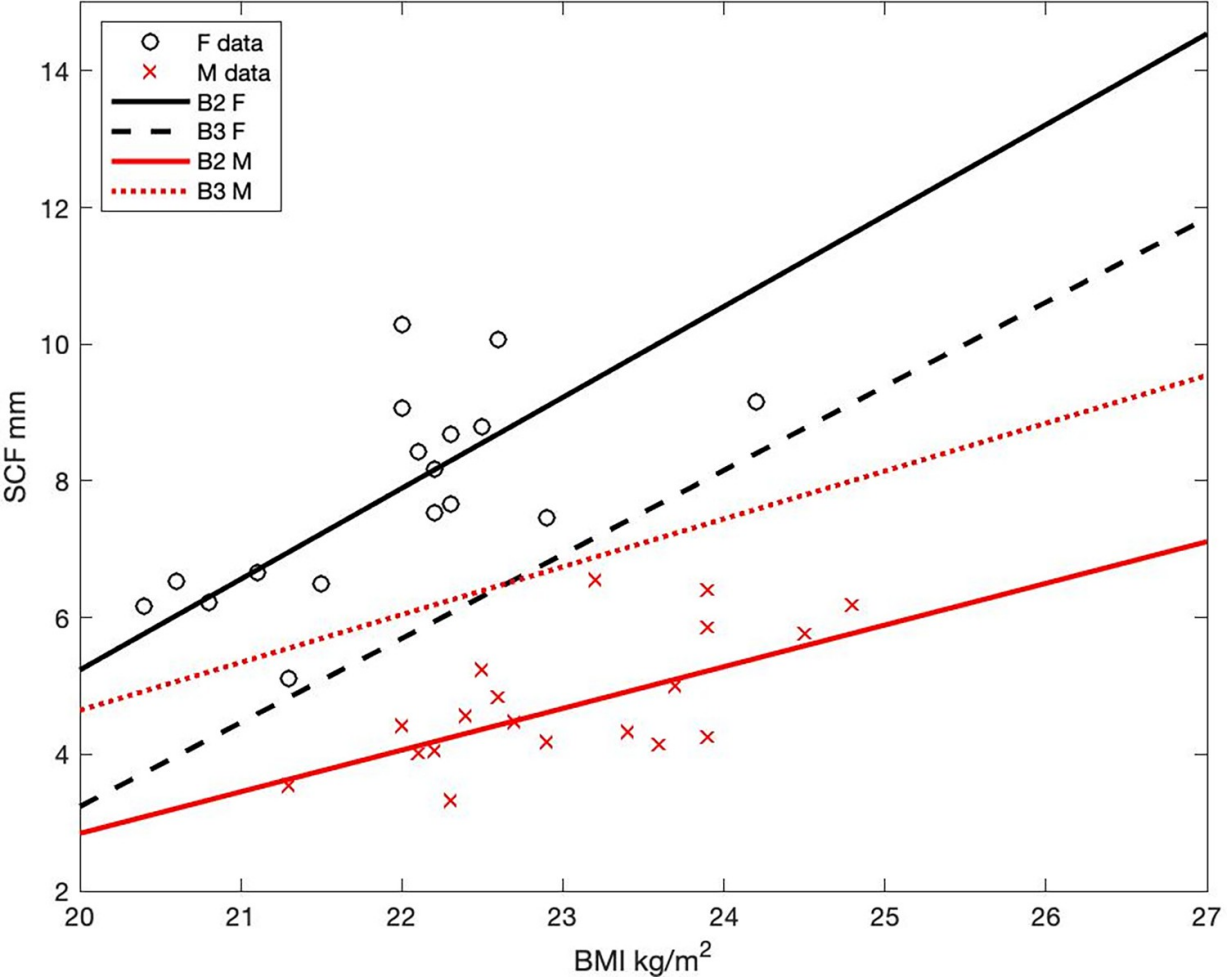
SCF-BMI relationship. Scatter plot and adopted linear relationships between body mass index, in kg/m^2^ (BMI) and subcutaneous fat layer thickness, in mm (SCF). Black circles and red Xs, are female and male data respectively. Solid, dashed lines represent the relationship used for body types B2 and B3 respectively. Note that the B3 F curve underestimates the lowest BMI-SCF relationship seen on the data (black circle at BMI~21.1, SFC~4,8) with the opposite being the case for B3 M (red X at BMI~23.1, SFT~6).

To evaluate the effect of body size on trip energy balance, *EB* is estimated for female and male bodies of different height (H), mass (BM), and subcutaneous fat layer thickness (SCF). Three body types are evaluated for each sex. Body types 1 (B1) are based on morphological data from Polynesian populations [13] (S1 Table in S1 File). Body types 2 and 3 (B2, B3) have the same height as B1 with weight increased in order to generate a two-unit gain in body mass index (BMI = BM/[H/100]^2^). B2 and B3 differ in their SCFs. In both cases, biometric data from 891 athletes covering 21 sport modalities [53] are used to generate linear relationships between BMI and SCF, and these are adopted to estimate SFC from BMI (Fig 2).

Female SCF is more sensitive to BMI than male SCF, leading to statistically significant differences in the *EB* response to changes in BMI among the sexes. To increase the robustness of conclusions based on this difference, SCF in the female B3 body type is based on the least sensitive SCF-BMI relationship seen in the data, while the opposite is done for SCF estimated for the male B3 body type (Fig 2).

#### EB estimates

**Fig 3:**
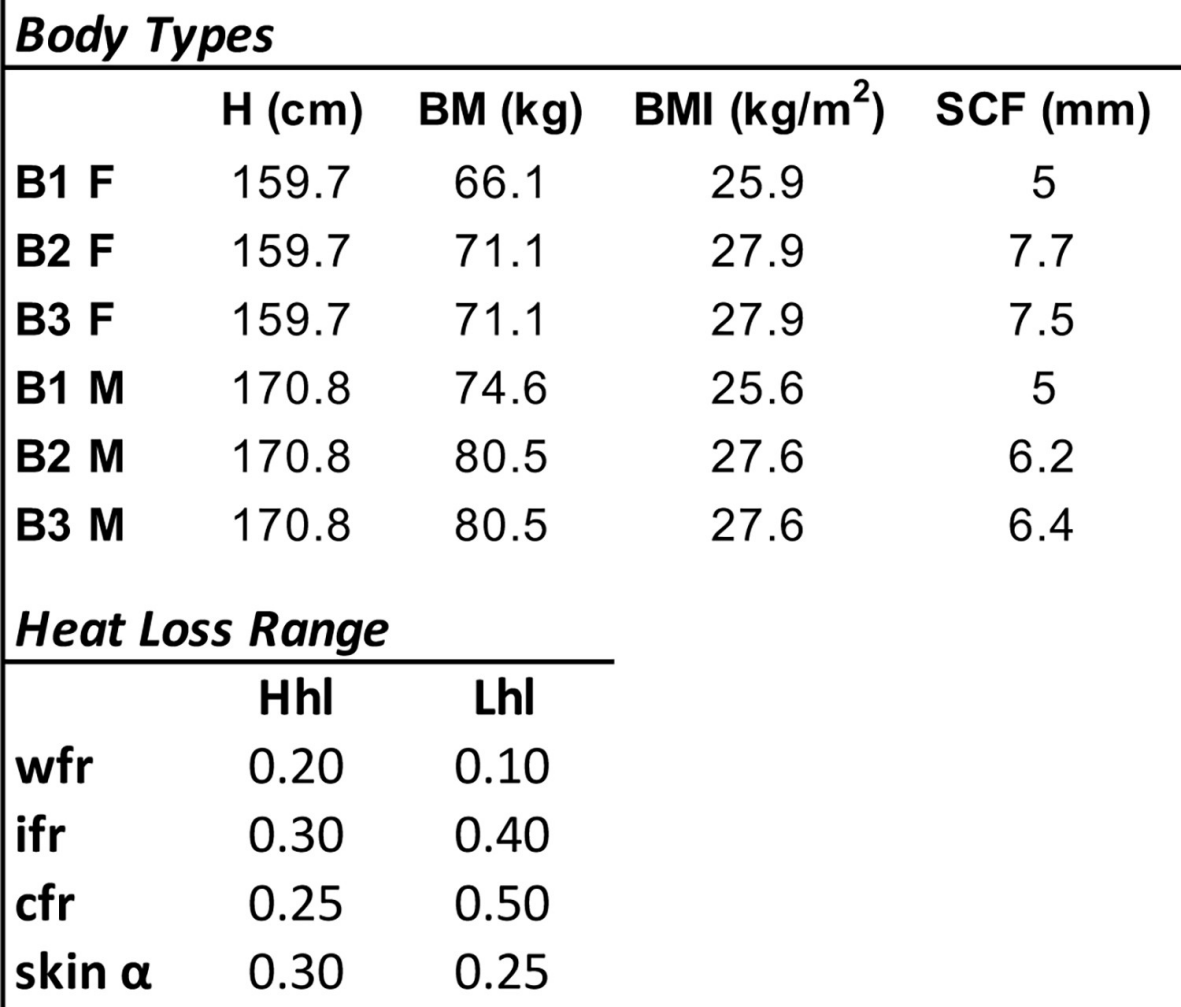
EB model parameters.

Hourly trip *EB* is calculated by averaging the environmental parameters experienced by all eight vessels taking a particular trip (i.e., vessels with same destination and departure date) meaning results are analyzed for every departure day and not specific vessels. *EB* is estimated for the six body types described in the section above (Fig 3, top). To bracket uncertainty associated with *EB* estimates, high (Hhl) and low (Lhl) net heat loss *EB* values are generated. Estimates vary in adopted skin reflectivity and on three parameters that indicate the level of exposure to the elements: fraction of the body that remains wet, illuminated by the sun, and covered by clothing (Fig 3, bottom). High and low heat loss estimates are calculated for each of the six body types evaluated, resulting in 12 (6 female, 6 male) *EB* estimates per trip. Parameters associated with high and low heat loss rates are kept constant during each experiment. That is, *wrf*, *ifr*, *cfr* and skin α do not change with the seasons.

Top, characteristics of the three female (B1-3 F) and three male (B1-3 M) adopted body types. H, height; BM, body mass, BMI, body mass index; SCF, subcutaneous fat layer thickness. Bottom, parameters adopted for the high (Hhl) and low (Lhl) net heat loss *EB* estimates. Wfr, ifr, and cfr are fractions of the body that remain wet, illuminated by the sun and covered by clothing, respectively; skin α, skin reflectivity or albedo. All values in bottom portion of table are unitless.

## Results

### Trip environmental parameters

**Fig 4:**
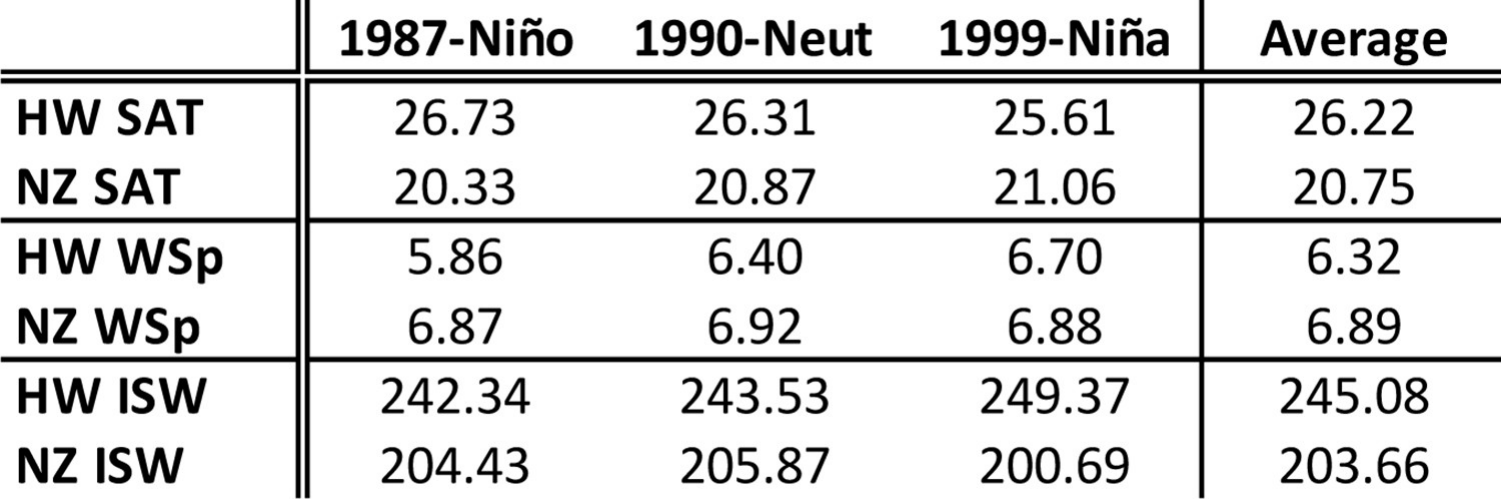
Environmental parameters. Annual and three-year averages of environmental parameters adopted by the *EB* model experienced by voyagers during Hawaii, HW, and New Zealand, NZ, trips. SAT, surface air temperature, in ˚C; WSp, wind speed, in m/s; ISW, incoming shortwave radiation, in W.

**Fig 5:**
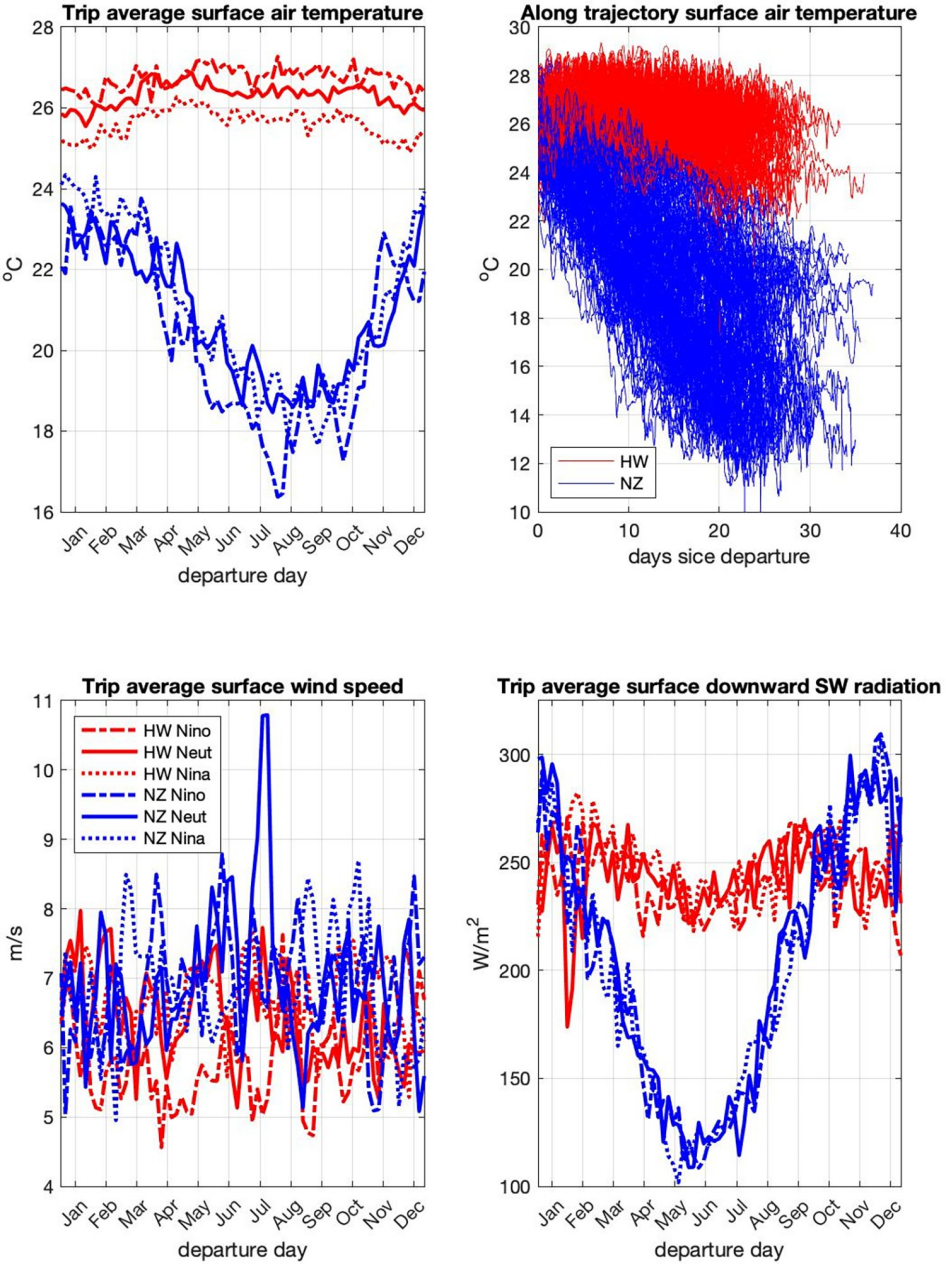
Environmental parameters adopted by *EB* model experienced by voyagers during trips. Top left, mean trip surface air temperature; top right, along-trajectory surface air temperature for all trips; bottom left, mean trip surface wind speed; bottom right, mean trip incoming shortwave radiation flux. HW and NZ, trips to Hawaii and New Zealand. Nino, Neut, and Nina represent El Niño, neutral, and La Niña conditions, that is, data from years 1987, 1990, and 1999 respectively.

The results of our simulations indicate that trips to Hawaii (~23.5 days) and New Zealand (~25.5 days, S2 Fig in S1 File) have similar average duration but distinct along-route environmental conditions. For most trips, travelers to New Zealand face lower temperatures, stronger winds, and weaker solar radiation fluxes (Figs 1 and 4). New Zealand-Hawaii temperature differences are larger during winter and the later portion of New Zealand trips, as these occur over higher latitudes (Fig 5). While New Zealand travelers receive less solar radiation than those going to Hawaii for most of the year, the reverse occurs between October and January.

### Energy balance

*EB* estimates provide insight on in-trip energetic demand of thermoregulation. Positive *EB* means traveler BMR more than compensates for body heat loss, and body temperature will tend to increase. If this persists for many hours, there will be a thermoregulatory response to avoid hyperthermia. The opposite is true for negative *EB*, with thermoregulatory costs increasing in order to match excess heat loss. Travelers would have to expend more energy through physiological processes such as shivering thermogenesis and BAT activation or behavioral processes such as physical activity in order to avoid hypothermia.

**Fig 6:**
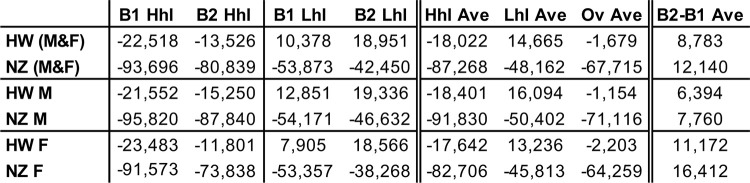
Aggregate *EB*. *aEB*, integral of whole trip *EB* results, in kcal, for all simulated trips. HW and NZ, Tahiti to Hawaii and New Zealand trips respectively; Hhl and Lhl, high and low net heat loss estimates, F and M, female and male; B1 and B2 body types 1 and 2; Hhl Ave and Lhl Ave, averages of both body types under high and low net heat loss estimates respectively; Ov Ave, overall average of all body types and heat loss estimates; B2-B1 Ave, average difference between body types B2 and B1 for both heat loss estimates. Positive values indicate lower heat loss for B2 bodies. Note that no information about B3 body types is present in this table.

*EB* rates are summed over whole voyages to generate *aggregate EB* (*aEB*, *kcal*), an estimate of the total trip caloric demand of thermoregulation that accounts for trip duration. The distinct along-route environmental conditions (Figs 1 and 4) cause large *aEB* differences between trips, with smaller values seen on voyages to New Zealand (Fig 6). Under the same heat loss assumptions—and when all simulated trips and both sexes are considered—*aEB* of New Zealand trips is about 3.3 to 4.8 times larger than that of Hawaii trips. Within estimate uncertainty, *aEB* results are unable to determine if travelers to HW need to thermoregulate by generating or losing heat. The larger BMI and thicker SCF of B2 type bodies results in lower heat loss and less negative *aEB* for the two destinations and both sexes, with larger reductions for female bodies and New Zealand trips (Fig 6*)*.

**Fig 7:**
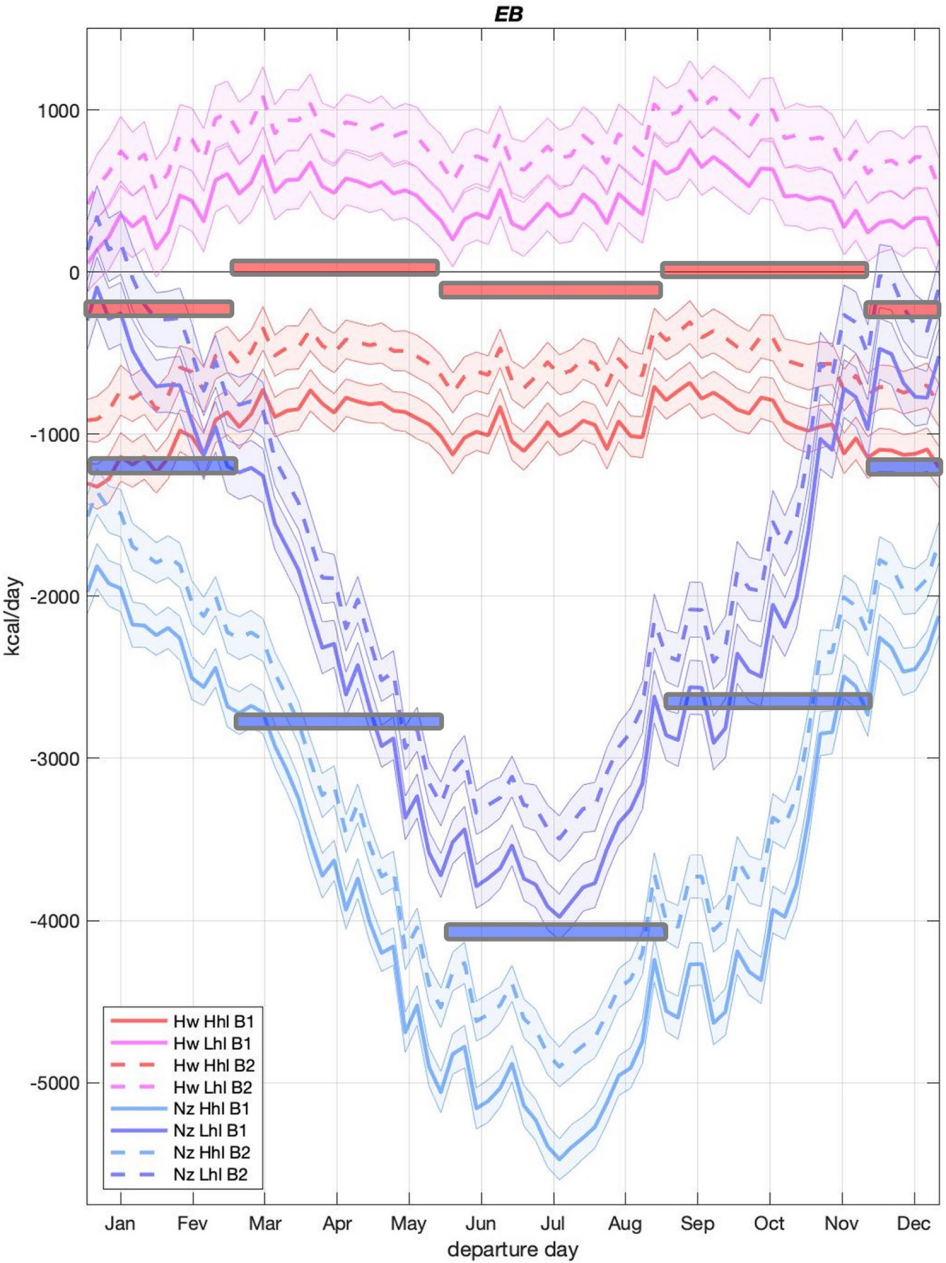
Whole trip *EB*. Averages, in kcal/day for all trips simulated for each departure day. HW and NZ are trips from Tahiti to Hawaii and to New Zealand. Hhl and Lhl are high and low heat loss estimates. B1 and B2 are body types. Shaded areas represent the 95% confidence interval of the mean. Horizontal bars represent seasonal averages for all of the shown New Zealand (blue) and Hawaii (red) trips. Note that no information about B3 body types is present in this table.

As with all-trips *aEB* results, departure-date-specific *EB* of trips to New Zealand are more negative, although differences between destinations decrease markedly during the Southern Hemisphere (SH) summer, reaching a seasonal minimum of ~965 kcal/day when averages of all heat loss estimates and body types are considered (Fig 7). This is primarily driven by warmer temperatures and greater insolation along New Zealand SH summer trips, with a smaller contribution from decreases in temperature and insolation along Hawaii trajectories, as these experience winter conditions in the Northern Hemisphere (Fig 5). Comparisons between same body types and heat loss assumptions show that *EB* differences remain statistically significant throughout the year with the exception of differences between low heat loss estimates during an approximately two-week window in early January (Fig 7) when along-trajectory air temperature differences are at their minimum. In addition, trips to New Zealand experience both higher insolation and lower wind speeds than those to Hawaii (Fig 5), which is different from all other periods of the year.

Results show that the energetic demands of trips to New Zealand are significantly larger than those to Hawaii, but what do these differences mean to the travel experience? Examples below aim to contextualize this *EB* difference in terms more directly related to trip effort. We take the conservative approach to focus on SH summer trips when EB differences between trips to New Zealand and Hawaii are minimal.

Based on SH summer *EB* averages (Fig 7), each traveler to New Zealand would require an extra 965 kcal/day for thermoregulation. If such deficit were completely compensated by use of energy reserves in the form of fat, individuals going to New Zealand would lose an extra 107.2 g of fat per day, or an extra 2.68 kg at the end of a 25-day trip, the simulated average duration. If the difference was solely compensated by consumption of muscle mass, whole trip extra weight loss would be 6.03 kg (13.3 lbs). Assuming no energy reserves are used, and all extra calories related to the *EB* difference came from food consumption, this would demand the additional daily ingestion, for example, to be either ~965 g (2.1 lbs) of fish, ~680 g (1.5 lbs) of cooked taro root, or ~574 g (1.3 lbs) of boiled breadfruit seeds (caloric contents from USDA [54]) or some combination thereof. Physical activity is one way to generate heat. For temperature of about 12˚C, ~11.6% of calories spent on physical activity go towards heating the body [3, 55]. Assuming this holds for the warmer temperatures of New Zealand journeys (Fig 5) and that all of the extra energy for thermoregulation comes from this source (i.e., excluding shivering and BAT), generating enough heat to cover the *EB* difference would involve individuals spending an additional 8,310 kcal/day performing physical activities. The above examples are mere “translations” of the *EB* difference into other quantities and are not presented as actual strategies.

**Fig 8:**
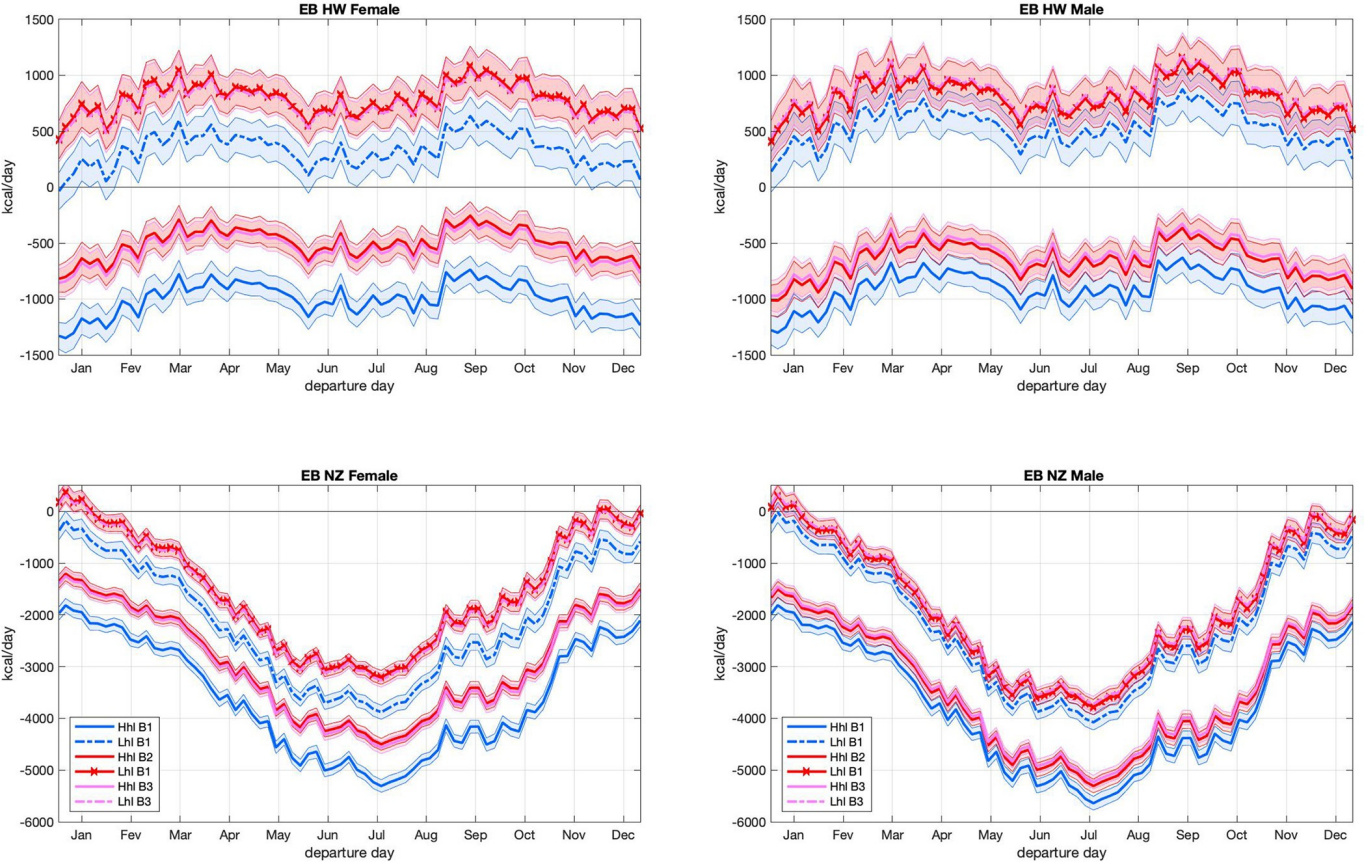
Effects of body type and sex on *EB*. *EB* in kcal/day, for different body types and heat loss estimates averaged by departure day. Left, female; right male; top, Hawaii; bottom New Zealand. Hhl and Lhl are high and low heat loss estimates. B1, B2, and B3 are body types. Shaded areas represent the 95% confidence interval of the mean.

The advantages conferred by the larger and better insulated B2 body type influence thermoregulation of all trips, with larger differences seen under conditions of greater heat loss and negative *EB* (Fig 7). These body-dependent differences are larger for females, and in fact not significant (95% confidence limit) for male bodies (Fig 8). This is in part due to SCF estimates used in female B2 types being more sensitive to changes in BMI than SCF estimates adopted by male B2 types (Fig 2). Body type B3—where the sensitivity of SFC to BMI is decreased for females and increased for males (Fig 2)—is adopted to test the robustness of results to changes in the sex-dependent BMI-SCF relationship. The inclusion of the B3 body type did not change the conclusions coming from the B1-B2 comparisons (Fig 8).

## Discussion and conclusions

Data collected throughout the 20^th^ century show that Polynesians of both sexes are about 5% taller than tropical Pacific islanders occupying areas to the west of the Lapita expansion region. They also have larger body mass, with females being about 31%, and males 24% heavier than populations to their west [13]. Selection for larger bodies under thermally demanding oceanic conditions has been suggested as a potential cause for this size difference [13] and this hypothesis is corroborated by the higher, or more positive, *EB* seen for larger bodies in the present results. Thermoregulatory gains associated with increase in body size is larger for females than for males. While our analysis cannot be used for definite conclusions about this difference, it is interesting to note that, compared to non-Polynesian islanders, Polynesian females are relatively larger than males.

These body-dependent differences between sexes may be reflective of sexual dimorphism in human fat distribution patterns. Females have greater body fat percentages than males and accumulate more fat in the thighs and hips, while males have more internal visceral fat [56]. Females have more efficient thermoregulation in hot and humid environments while males fare better in hot and dry environments [57]. In cold environments, greater fat percentages provide females with greater energetic stores relative to males, which provide a buffer in a negative energy balance [52]. Greater central adiposity patterns in females may protect them against the cold as observed in cold adapted Indigenous populations [58, 59]. Preliminary studies suggest females have greater BAT activity compared to males, highlighting the potential for greater BAT-induced thermoregulatory protection in females [60].

Present results show that while trips from Tahiti to Hawai‛i and Tahiti to New Zealand have similar duration, travelers to New Zealand are exposed to harsher environmental conditions, leading to significantly larger in-trip energetic demands. This makes voyages to New Zealand and other higher-latitude islands riskier and in need of more complex planning in terms of managing time of departure, protection from the elements, and food procurement and provisioning. For both tested destinations, travelers with larger body size exhibit lower modeled heat loss and hence obtain an energetic advantage, with gains being larger for females. Although this agrees with the observed larger bodies of Polynesian populations, the mechanistic links between *EB* and body would size require further analysis.

Overall, our research helps to answer some fundamental questions regarding the challenges that Polynesian voyagers faced when colonizing the Pacific which stretches across millions of square kilometers of open ocean. Why are there stark chronological disparities between when some archipelagos were settled versus others? Why were some islands colonized but abandoned at European contact (the so-called ‘mystery islands’)? How can we better estimate success rates of voyages, particularly between tropical and more temperate regions, knowing that not all of them were successful?

The region’s colonization history is replete with both exceptional and challenging voyages across the ocean. These range from the first forays into Remote Oceania with Lapita groups in the Southern Hemisphere and those settling western Micronesia (Palau, Mariana Is.) in the Northern Hemisphere between 3300–3000 BP. Later, pulses northward into central and eastern Micronesia (Chuuk, Pohnpei, Kosrae, Kiribati, Marshall Is.) around 2000 BP, and the rapid dispersal c. 1200–1000 BP from Samoa and/or Tonga into East Polynesia after a 2000–1800 year “long pause” [11, 61, 62], 2016), represent more ambitious voyages that eventually reached the most remote islands on Earth. However, the underlying physiological challenges that voyagers would have faced when sailing from the tropics into temperate zones has never been explored quantitatively until now.

It is clear that navigational (wayfinding) techniques (e.g., sidereal compass) and boat construction (e.g., double hulled canoe, sail configurations) provided the means for Polynesians to sail long distances and often return home, but they did not guarantee successful passages. Our research demonstrates that energetic demands required to thermoregulate when moving from the tropics to temperate climates would have posed a significant obstacle for voyagers, particularly when traveling southward to New Zealand and nearby islands. The number of unsuccessful voyages will never be known, but it is likely that the greater fat content of East Polynesian voyagers, and their subsequent ability to thermoregulate more effectively, provided the necessary physiological advantages to overcome harsher and colder climates and help ensure survivability.

## Supporting information

S1 File Simulated trips and Pacific morphological data. Details of simulated trips including figures with sample trajectories, annual and interannual variability of average trip duration and speed. Table describing the morphological data adopted by the energy balance mode.
(DOCX)
