# Supplementary material for: Estimated energetic demands of thermoregulation during ancient canoe passages from Tahiti to Hawaii and New Zealand, a simulation analysis
Source: PLoS One. 2023 Jul 12;18(7):e0287290. doi: 10.1371/journal.pone.0287290 (PMC10337932; doi:10.1371/journal.pone.0287290)
Supplement: S1 File — Details of simulated trips including figures with sample trajectories, annual and interannual variability of average trip duration and speed. Table describing the morphological data adopted by the energy balance mode. (DOCX) [file pone.0287290.s001.docx]

**S1 Simulated Trips and Pacific Morphological Data**

Fig SI1 Samples of simulated trajectories
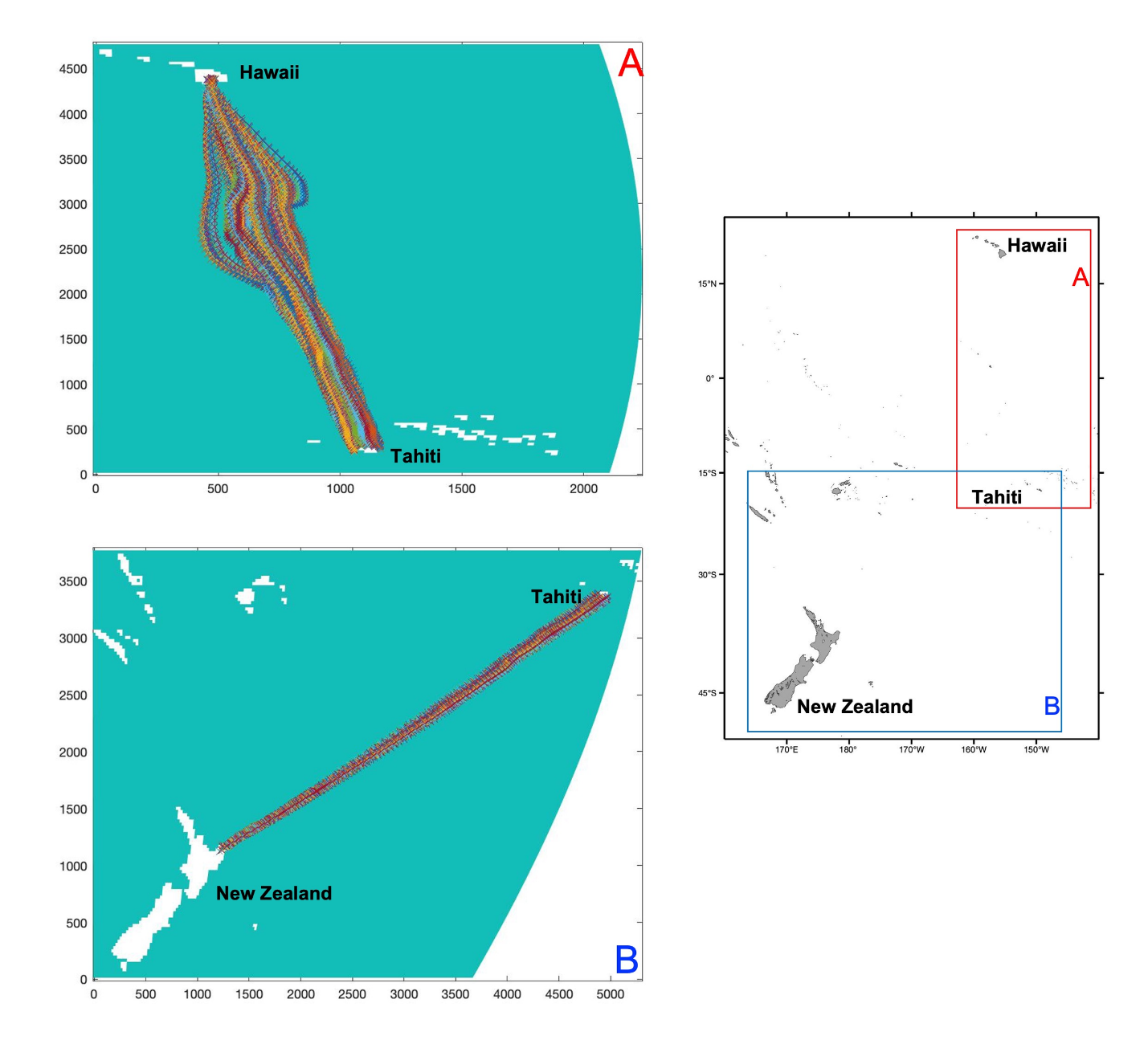
**Fig SI1:** Sample of Tahiti to HawIi’i, top left; and Tahiti to Te Ika-a-Māui, bottom left, sailing trip trajectories generated by the ocean voyaging model using input wind and currents from Jan 1990. Trajectory maps are plotted on distance coordinates (km from bottom right corner) and cover the approximate areas delineated on the lat-long coordinates map on the right.

Trip duration and vessel speed

Duration of trips to Hawaii range from ~18 to ~36 days and to New Zealand from ~15 to ~37 days. Trips to New Zealand tend, on average, to be two to three days shorter than those to Hawaii, the exception being the longer Hawaii trips simulated for 1987, under El Niño conditions (FigSI2 *)*. Arrival in Hawaii from Tahiti requires westward displacement, and trips under the weaker easterly trade winds associated with El Niño are, with 95% confidence, two to three days longer than the others, making them similar to New Zealand trips. While the same argument supports the observed shorter mean trips to Hawaii under La Niña (1990), the difference between neutral and La Niña duration averages is not statistically significant at 95% confidence. With trajectories less influenced by equatorial dynamics, duration of trips to New Zealand is not sensitive to ENSO variability. Mean Hawaii trip speeds range from ~2.5 kt to ~5.4 kt and those to New Zealand from ~2.5 kt to ~6.5 kt (FigSI3 ). As expected, given the discussed differences in duration, slightly faster annually averaged speeds are simulated for the neutral and La Niña Hawaii trips.

Fig SI2 Trip duration

**
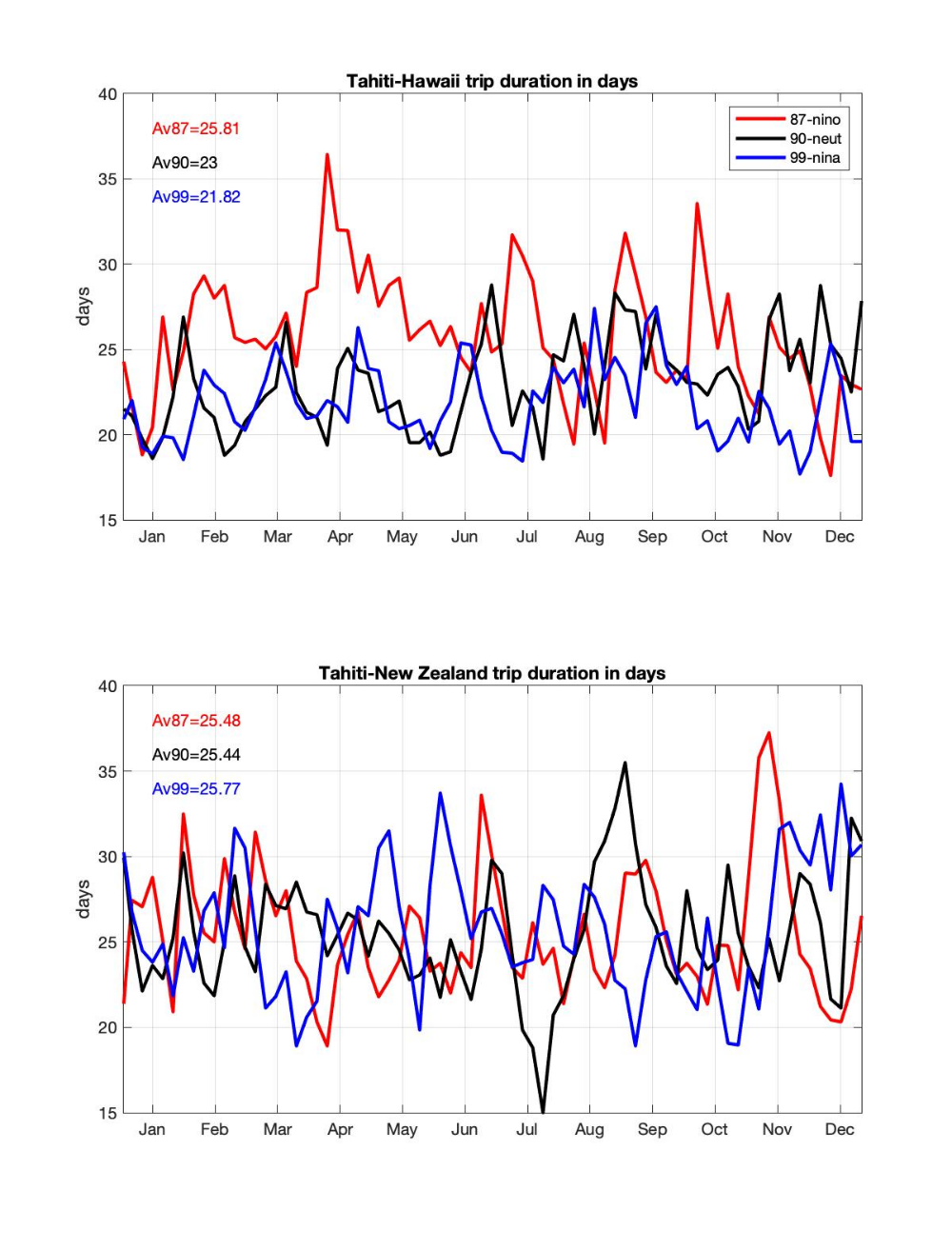
**

**Fig SI2:** Trip duration, in days, averaged for each of the 73 departure days. Red, black, and blue refer to trips simulated using input wind and currents from 1987 (El Niño), 1990 (Neutral), and1999 (La Niña) respectively. Av** is the average duration for all trips started in a particular year. Top, Tahiti to Hawaii trips; bottom, Tahiti to New Zealand trips.

Fig SI3 Trip speed

**
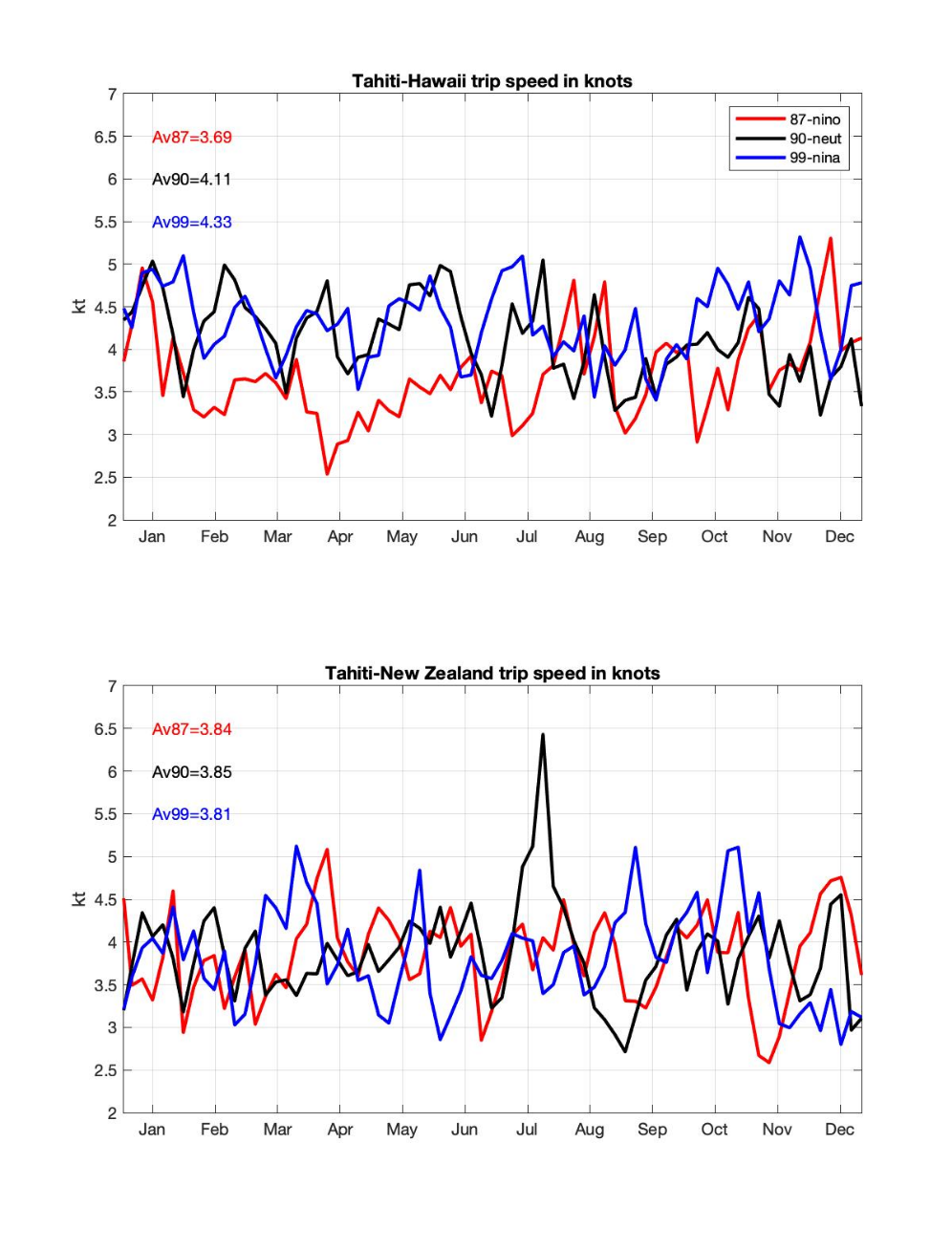
**

**Fig SI3:** Like S2 Fig but for average trip vessel speed in knots

Table SI1 Morphological data

**Table SI1:** Details of the morphological data adopted by the energy balance model. Average weight (kg) and height (cm) used for the overall mean Polynesian values adopted by the energy balance model., Std Dev is standard deviation. All values from [13]
